# Apoptosis is associated with triacylglycerol accumulation in Jurkat T-cells

**DOI:** 10.1038/sj.bjc.6600188

**Published:** 2002-03-18

**Authors:** N M S Al-Saffar, J C Titley, D Robertson, P A Clarke, L E Jackson, M O Leach, S M Ronen

**Affiliations:** Cancer Research UK Clinical Magnetic Resonance Research Group, Royal Marsden NHS Trust, Downs Road, Sutton Surrey SM2 5PT, UK; Cancer Research UK Centre for Cancer Therapeutics, Royal Marsden NHS Trust, Downs Road, Sutton Surrey SM2 5PT, UK; Electron Microscopy Unit, Institute of Cancer Research, Royal Marsden NHS Trust, Downs Road, Sutton Surrey SM2 5PT, UK

**Keywords:** MRS, apoptosis, Fas, triacylglycerols

## Abstract

Magnetic resonance spectroscopy is increasingly used as a non-invasive method to investigate apoptosis. Apoptosis was induced in Jurkat T-cells by Fas mAb. ^1^H magnetic resonance spectra of live cells showed an increase in methylene signal as well as methylene/methyl ratio of fatty acid side chains at 5 and 24 h following induction of apoptosis. To explain this observation, ^1^H magnetic resonance spectra of cell extracts were investigated. These demonstrated a 70.0±7.0%, 114.0±8.0% and 90.0±5.0% increase in the concentration of triacylglycerols following 3, 5 and 7 h of Fas mAb treatment (*P*<0.05). Confocal microscopy images of cells stained with the lipophilic dye Nile Red demonstrated the presence of lipid droplets in the cell cytoplasm. Quantification of the stained lipids by flow cytometry showed a good correlation with the magnetic resonance results (*P*⩾0.05 at 3, 5 and 7 h). ^31^P magnetic resonance spectra showed a drop in phosphatidylcholine content of apoptosing cells, indicating that alteration in phosphatidylcholine metabolism could be the source of triacylglycerol accumulation during apoptosis. In summary, apoptosis is associated with an early accumulation of mobile triacylglycerols mostly in the form of cytoplasmic lipid droplets. This is reflected in an increase in the methylene/methyl ratio which could be detected by magnetic resonance spectroscopy.

*British Journal of Cancer* (2002) **86**, 963–970. DOI: 10.1038/sj/bjc/6600188 www.bjcancer.com

© 2002 Cancer Research UK

## 

Apoptosis is a genetically controlled pathway of cell death which exhibits distinct morphological features. Accruing evidence shows that apoptosis and genes associated with it have a significant effect on malignant phenotype. Moreover, it is now well documented that most cancer therapeutics kill cells *in vitro* and *in vivo* through the activation of apoptotic cell death (Ellis *et al*, 1997; Hickman, 1996). As a result intensive research has been focused on understanding the genetic basis of apoptosis and the development of novel therapies that are designed to launch specifically the apoptotic machinery of the cell. The development of methods for the detection of apoptosis would also be useful as early markers for response in treated tumours.

Magnetic resonance spectroscopy (MRS) has been used in several studies concerned with apoptotic cell death (Bhakoo and Bell, 1997; Williams *et al*, 1998; Ronen *et al*, 1999). In investigating cell death in several systems including Fas-induced apoptosis in Jurkat T-cells, Blankenberg and co-workers have found that the increase in methylene/methyl (CH_2_/CH_3_) signal intensity ratio of fatty acid side chains correlates with the onset of apoptosis, suggesting that ^1^H MRS could be used to detect and quantify apoptotic cell death *in vitro* (Blankenberg *et al*, 1996, 1997). The authors speculate that the increase in this ratio is due to increased mobility of plasma membrane lipids. In contrast, an *in vivo* investigation of gene therapy-induced apoptosis showed an increase in both CH_2_ and CH_3_ resonances with no significant alteration in their ratio (Hakumaki *et al*, 1999). In this case the alterations in ^1^H MR spectra were reported to be associated with an accumulation of polyunsaturated fatty acids. ^13^C and ^31^P MRS have been applied to investigate the nature of the accumulated lipids and the mechanism underlying changes in lipid and fatty acid metabolism during apoptosis. Treatment of KB cells with Miltefosine (HePC) resulted in a decrease in phosphatidylcholine (PtdCho) and an increase in the neutral lipids triacylglycerols (TAGs) and diacylglycerols (DAGs) as well as fatty acid synthesis, indicating a possible impairment of the DAG pathway during HePC-induced apoptosis (Engelmann *et al*, 1996). An inhibition of PtdCho synthesis was also observed in HL-60 cells undergoing apoptosis induced by a variety of cytotoxic drugs (Williams *et al*, 1998).

In this study we investigated ^1^H MR spectra of intact cells as well as cell extracts of control and apoptotic Jurkat T-cells. Combined with ^31^P MRS, flow cytometry and confocal microscopy we have tried to determine the nature, origin and mechanism of lipid changes associated with apoptosis. We demonstrate that in intact cells, an increase in CH_2_/CH_3_ ratio occurs in the early stage of Fas-induced apoptosis and is associated with an early accumulation of TAGs mostly in the form of cytoplasmic lipid droplets.

## MATERIALS AND METHODS

### Cell culture and treatment

Jurkat (human acute lymphoblastic T cell leukaemia, clone E6.1) cells were obtained from ECACC (UK) and grown in RPMI 1640 medium supplemented with 10% (v v^−1^) heat-inactivated foetal calf serum (FCS), 2 mM L-glutamine, 50 U ml^−1^ penicillin and 50 μg ml^−1^ streptomycin (Gibco, UK) in an atmosphere of 5% CO_2_ in air at 37°C. For biological and MRS investigations of apoptosis, Jurkat cells (10^6^ cells ml^−1^) were stimulated with 100 ng ml^−1^ mouse monoclonal IgM anti-human Fas mAb, clone CH-11 (TCS Biological, UK).

### Cell cycle analysis (CCA)

Cells (10^6^) were washed in phosphate buffered saline (PBS), fixed in 70% ethanol, incubated with 100 μg ml^−1^ RNase A and 40 μg ml^−1^ propidium iodide (PI) (Sigma, UK) in citrate-buffered saline for 30 min at 37°C (Hotz *et al*, 1994). DNA histograms were generated by fluorescence activated cell sorting (FACS) analysis using an EPICS Elite ESP cell sorter (Beckman-Coulter, High Wycombe, UK) at 488 nm. Data were plotted and analysed using WinMDI version 2.7 and Cylched flow cytometry application software (University of Wales College of Medicine, Cardiff based activities, UK).

### Mitochondrial transmembrane potential (ΔΨ_m_)

Mitochondrial ΔΨ_m_ was measured by incubating live cells (10^6^) at 37°C with 1 ml of PBS solution containing 50 nM of 3,3′-dihexyloxacarbocyanine iodide (DiOC_6_ (3); Molecular Probes, UK) for 30 min. Samples were immediately analysed by FACS on an EPICS Elite ESP cell sorter. Data were analysed using WinMDI version 2.7 software.

### MRS of intact cells

Live cells (2×10^8^) were washed twice with PBS-D_2_O and suspended in 400 μl of PBS-D_2_O in a standard 5 mm NMR tube. A 410 μl capillary containing 0.075% TSP (Sigma, UK) in PBS-D_2_O served as an external reference (0.0 p.p.m.). ^1^H MR spectra were acquired at room temperature on a 250 MHz Bruker spectrometer using a 60° flip angle, a 1 s relaxation delay, spectral width of 20 p.p.m. and 64 K data points. HDO resonance was suppressed by presaturation. Spectra were plotted and analysed using MestRe-C version 1.5.1 software (http://qobrue.usc.es/jsgroup/MestRe-C/MestRe-C.html). CH_2_ and CH_3_ contents were determined by integration and normalised relative to TSP and cell number. Lactate contribution to the CH_2_ resonance was subtracted following deconvolution (Barba *et al*, 2001) using xedplot software (Bruker).

### MRS of cell extracts

At different time points, cell extracts were obtained from 1–2×10^8^ control or treated cells, using a modification of the dual phase extraction method (Ronen *et al*, 1999). This method separates aqueous metabolites, lipid metabolites and proteins. ^31^P MR spectra of the lipid fraction of cell extracts were acquired at room temperature on a 400 MHz Bruker spectrometer using a 90° flip angle, a 7 s relaxation delay, spectral width of 20 p.p.m., and 32 K data points. For ^1^H MR analysis, lipid samples were resuspended in 700 μl of CDCl_3_ containing 0.03% TMS (Sigma, UK). ^1^H MR spectra were acquired at room temperature on a 600 MHz Bruker spectrometer using a 45° flip angle and a 2 s relaxation delay, spectral width of 12 p.p.m. and 32 K data points. To remove the broad peaks arising from relatively immobile components of lipid cell extracts and to enable quantification of TAG peaks at 4.3 p.p.m., ^1^H MR spectra of lipid extracts were also acquired using a Carr-Purcell-Meiboom-Gill (CPMG) pulse sequence 90°-(τ-180°-τ)_n_-acquire (τ=0.77 ms, *n*=128) and a 2 s relaxation delay. Spectra were plotted and analysed as above. Metabolite contents were determined by integration and normalised relative to internal standards and cell number.

### Nile Red staining

Unfixed cells (10^6^) or cells fixed for 1 h at room temperature with 1 ml of 4% paraformaldehyde/PBS pH 7.4, were stained with Nile Red as described previously (Greenspan *et al*, 1985). Briefly, cell pellets were rinsed once with RPMI 1640, incubated at room temperature with 1 ml of PBS solution containing 100 ng ml^−1^ of the fluorescent stain Nile Red (Sigma, UK) for at least 5 min. Co-staining with Nile Red and PI was obtained by permeabilising the fixed cells with 0.5% Triton X-100/PBS for 10 min, incubating at 37°C with RNase A for 30 min, staining cells with Nile Red as above and then staining cell pellets with PI for 2 min. Stained cells were mounted in a Vectorshield without DAPI (Vector Laboratories, USA), then viewed with a Leica TCS SP confocal microscope. Fixed cells were used in the case of confocal microscopy as immediate access to equipment was not always possible (no difference in neutral lipid localisation within cells was observed between fixed and live cells). Live cells stained with Nile Red were also analysed on an EPICS Elite ESP cell sorter. Data were analysed using WinMDI version 2.7 software.

### Statistics

Results are expressed as mean±s.d. and *n*⩾3 unless otherwise specified. Data were analysed using an unpaired two-tailed Student's standard *t*-test or ANOVA (Arcus Quickstat Biomedical) and differences were assumed to be significant when *P*<0.05.

## RESULTS

**Figure 1:**
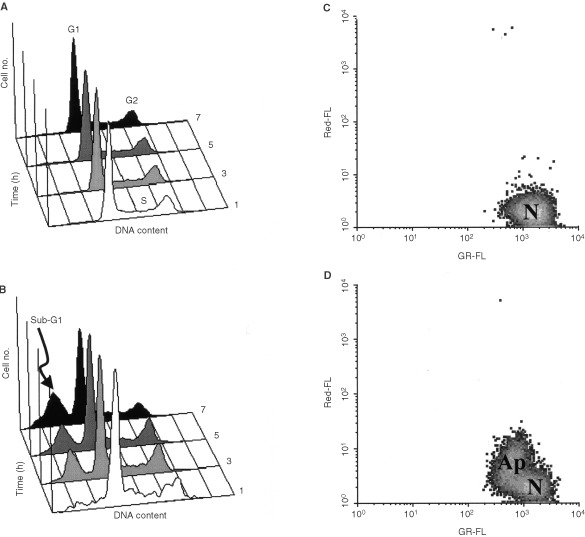
Cell cycle analysis by FACS of (**A**) Control, (**B**) Fas mAb treated (100 ng ml^−1^) Jurkat T-cells as a function of time. The presence of hypodiploid apoptotic cells is indicated by build up of the sub-G1 population. Analysis of mitochondrial ΔΨ_m_ of (**C**) Control, (**D**) Fas mAb treated (100 ng ml^−1^, 1 h) Jurkat T-cells by DiOC_6_(3) uptake. N=normal cells, strong green (DiOC_6_) log fluorescence (GR-FL), weak red (PI) log fluorescence (Red-FL); Ap=apoptotic cells, weak green (DiOC_6_) log fluorescence (GR-FL), weak red (PI) log fluorescence (Red-FL).

The human T-cell leukaemia ‘Jurkat’ were used as an *in vitro* model to study the metabolic changes that are detectable by ^1^H MRS in intact cells and cell extracts following Fas-induced apoptosis. The Fas system was chosen as a model system as it activates the two pathways described for apoptosis in general (Green, 2000). Treatment with the Fas mAb resulted in induction of apoptosis. Figure 1A,B

show the time course for the appearance of hypodiploid apoptotic cells detected as a sub-G1 population of cells following cell cycle analysis (CCA) by FACS. Apoptotic cells could be detected within 3 h of Fas mAb treatment. Loss of mitochondrial ΔΨ_m_ occurred within 1 h (Figure 1C,D).

**Figure 2:**
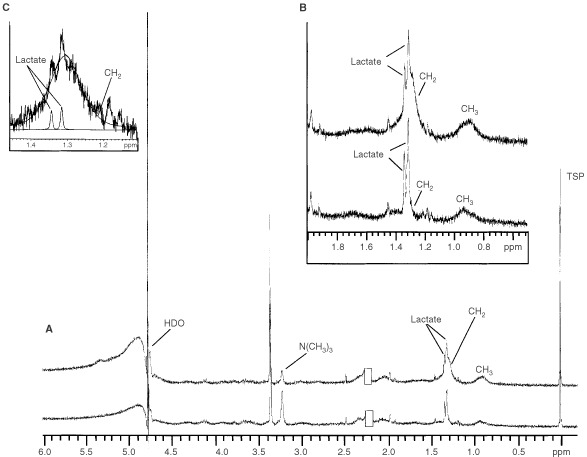
(**A**) ^1^H MR spectra of control (bottom) and 24 h Fas mAb treated (100 ng ml^−1^) (top) intact Jurkat T-cells, (**B**) An expanded region of the ^1^H MR spectra showing the increase in CH_3_ and CH_2_ resonances following induction of apoptosis, (**C**) An example of the deconvolution carried out to quantify changes in the CH_2_ peak. Spectra are the result of 128 scans plotted with line broadening of 0.1 Hz and are acquired using similar cell numbers for control and treated cells (residual acetone @ 2.22 p.p.m. was removed from plots).

MRS was used to identify the metabolic changes occurring following Fas-induced apoptosis. ^1^H MR spectra of intact Jurkat cells were acquired following 5 and 24 h of Fas mAb treatment. Spectra of treated cells showed a 314.0±107.0% and 603.0±242.0% (*P*<0.02) increase in CH_2_ signal as well as a 224±104.0% and 196±69.0% (*P*<0.04) increase in CH_2_/CH_3_ ratio at 5 and 24 h respectively. Interestingly, an increase in the CH_3_ resonance was only observed following 24 h treatment relative to control (up to 246.0±67.0%, *P*<0.003) (Figure 2

). However, using both CCA and morphological analysis as well as trypan blue exclusion we found that the majority of 24 h treated cells had undergone secondary necrosis. In contrast, cells treated for 5 h displayed clear apoptotic morphology. Consequently, further investigations of lipid metabolism during apoptosis concentrated on cells treated with Fas mAb for up to 7 h when no secondary necrosis could be observed.

**Figure 3:**
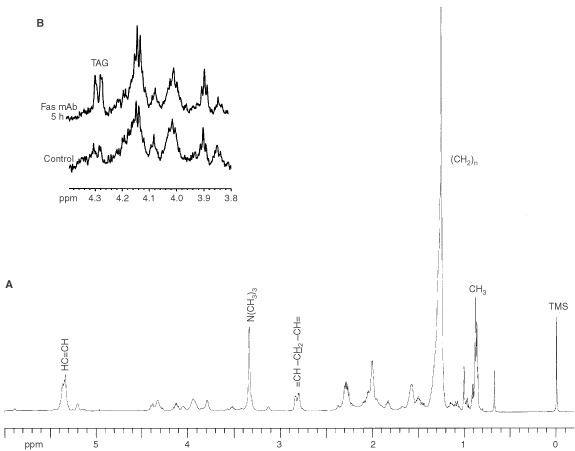
(**A**) ^1^H MR spectrum of the lipid fraction of Jurkat T-cell extracts, (**B**) An expanded region of the CPMG ^1^H MR spectra of the lipid fractions of control and Fas mAb treated (100 ng ml^−1^) Jurkat T-cell extracts. This illustrates the increase in the triacylglycerol (TAG)-specific peak around 4.3 p.p.m. Spectra are the result of 128 scans plotted with line broadening of 1 Hz.

In order to remove proteins and other aqueous metabolites that could contribute to the CH_3_ and CH_2_ peaks in ^1^H MR spectra (Kauppinen *et al*, 1993; Di Vito *et al*, 2001), further investigations of lipid metabolism were performed by monitoring lipid cell extracts at different time points. An example of the lipid phase ^1^H MR spectrum of control Jurkat cell extracts is shown in Figure 3A

. No significant change was found in the CH_2_ and CH_3_ peak areas or in the CH_2_/CH_3_ ratio of the treated cell extracts relative to control. Furthermore, there were no detectable changes in treated *vs* control cells in the areas of the peaks resonating at 5.4 and 2.8 p.p.m., which are assigned to the vinyl and bis-allylic protons of mobile polyunsaturated fatty acids. However, analysis of ^1^H MR spectra run under the CPMG pulse sequence showed an increase in the peaks resonating at 4.3 p.p.m. in treated cell extracts relative to controls (Figure 3B). Literature assignments show that this peak results from the interactions between protons at carbon 1 and 3 of the glycerol backbone of TAG with their geminal (4.17 p.p.m.) and vicinal (5.25 p.p.m.) protons giving rise to a doublet of doublet resonances which is specific to TAG (Casu *et al*, 1991). Assignments were confirmed by 2D COSY ^1^H MRS (data not shown). As illustrated in Table 1

**Table 1:**
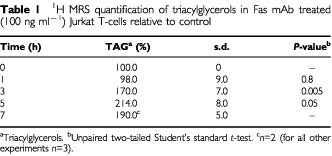
^1^H MRS quantification of triacylglycerols in Fas mAb treated (100 ng ml^−1^) Jurkat T-cells relative to control

, quantification of the amount of TAG showed a significant increase following treatment with Fas mAb (up to 214.0±8.0% at 5 h, *P*<0.05).

**Figure 4:**
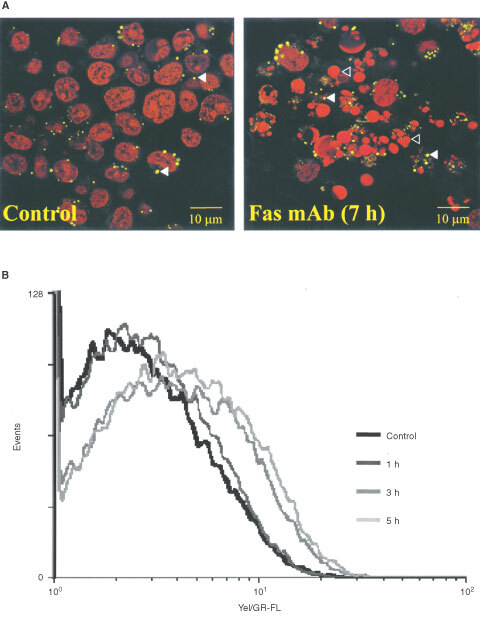
(**A**) Confocal microscopy images of control and Fas mAb treated (100 ng ml^−1^) Jurkat T-cells co-stained with the lipophilic dye Nile Red and PI. The yellow represents Nile Red stained neutral lipid droplets, examples are indicated by full arrowheads. Examples of apoptotic bodies are indicated by open arrowheads. (**B**) Yellow/Green log fluorescence (Yel/GR-FL) in Nile Red stained Jurkat T-cells following treatment with Fas mAb (100 ng ml^−1^) as a function of time. This illustrates the build up of neutral lipids in treated cells as detected by FACS.

Confocal microscopy images of cells stained with Nile Red – a lipophilic dye that serves as a sensitive stain for the detection of neutral lipids (Greenspan *et al*, 1985) – demonstrated the presence of lipid droplets in the cell cytoplasm. Confocal microscopy images of cells co-stained with PI and Nile Red further confirmed the onset of apoptosis which was indicated by the presence of apoptotic bodies in treated cells compared to controls (Figure 4A

). It also showed that cytoplasmic lipid droplets within the treated population have increased in size and fluorescence intensity.

Flow cytometry was used for an accurate quantification of mobile lipid content per cell. This showed that fluorescence emitted by Nile Red stained neutral lipids increased as a function of time following treatment with Fas mAb (Figure 4B). As illustrated in Table 2

**Table 2:**
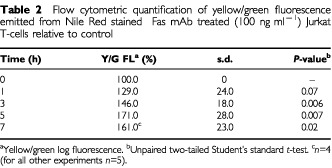
Flow cytometric quantification of yellow/green fluorescence emitted from Nile Red stained Fas mAb treated (100 ng ml^−1^) Jurkat T-cells relative to control

, quantification of the mean fluorescence emitted per cell showed a significant increase following the induction of apoptosis (up to 171.0±28.0% at 5 h, *P*<0.01) relative to control (*n*=5). This increase showed a good correlation with the amount of TAGs measured by ^1^H MRS (*P*⩾0.05 ^1^H MRS *vs* FACS for each time point). Furthermore, the accumulation of neutral lipids was first detected by FACS at 1 h (129%±24.0%, *P*<0.08). This is as early as the loss of mitochondrial ΔΨ_m_, a known early event in the apoptotic cascade (Figure 1C,D).

To investigate if neutral lipid accumulation in our model is caused by cell cycle arrest as reported in some systems (Delikatny *et al*, 1996; Barba *et al*, 1999), we used FACS analysis to quantify the DNA content of control and treated cells. Cell cycle analysis showed that up to 7 h, Fas mAb treatment has only resulted in the appearance of the subG1 peak indicative of apoptosis. When the distribution of cells in different phases of the cell cycle was determined, control cells and the non-apoptotic fraction of Fas-treated cells had very similar distributions. Both in control and treated cells the distribution ranged between 55–58% in G1, 30–35% in S and 10–12% in G2 phases (*n*=4) over different time points. Moreover, the number of Fas-treated cells was comparable to control cells throughout the treatment time, ruling out any cytostatic effect of Fas mAb.

**Figure 5:**
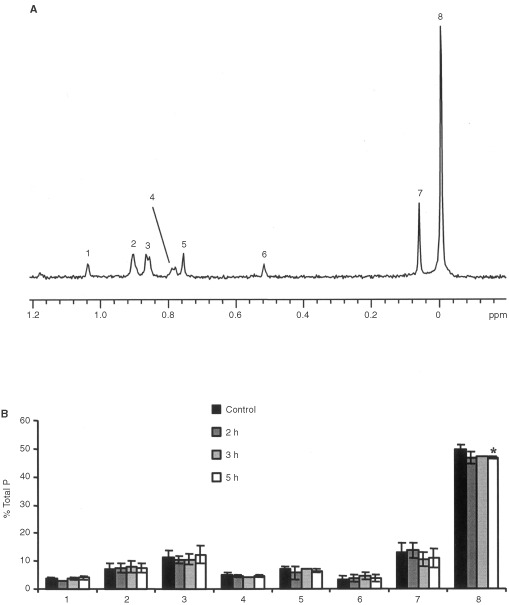
(**A**) ^1^H decoupled ^31^P MR spectrum of the lipid fractions of Jurkat T-cell extracts (1=cardiolipin, 2=plasmalogen phosphatidylethanolamine, 3=phosphatidylethanolamine, 4=phosphatidylserine, 5=sphingomyelin, 6=phosphatidylinisitol, 7=plasmalogen phosphatidylcholine, 8=phosphatidylcholine). Spectrum is the result of 1280 scans plotted with line broadening of 0.1 Hz. (**B**) Time course for the changes in phospholipid content of Jurkat T-cells showing the drop in phosphatidylcholine levels following Fas mAb treatment (100 ng ml^−1^) (* *P*<0.01).

The ^31^P MR spectrum of the lipid fraction of control Jurkat T-cells is shown in Figure 5A

. The only change observed was in PtdCho which dropped from 50±2.0% of the total phospholipid content in control to 47±0.5% following 5 h of treatment (*P*<0.01) (Figure 5B).

## DISCUSSION

Using ^1^H MRS, we have investigated spectra of intact cells as well as cell extracts of control and apoptotic Jurkat T-cells. Combined with ^31^P MRS, flow cytometry and confocal microscopy we have tried to determine the nature, origin and mechanism of lipid changes associated with apoptosis.

In agreement with previous reports (Blankenberg *et al*, 1996, 1997), an increase in the CH_2_/CH_3_ ratio was observed in ^1^H MR spectra of intact apoptotic cells following 5 and 24 h of Fas mAb treatment. In addition, an increase in both CH_2_ and CH_3_ resonances was also observed in line with other work (Hakumaki *et al*, 1999). However this increase was only observed following 24 h of treatment, whereas at 5 h an increase in CH_2_ was observed but CH_3_ remained constant. This might be due to the fact that at the late 24 h time point, the majority of the cells had undergone secondary necrosis leading to loss of cell membrane integrity. Therefore to investigate apoptosis-specific changes, we have concentrated on investigating changes in lipid metabolism during the first 7 h following Fas mAb treatment when the majority of cells displayed apoptotic morphology and 99.0% of the cells had retained membrane integrity.

A change in the ^1^H MRS lipid profile can result from an increase in the mobility of lipid fatty acid chains as a result of a change in cellular localisation, membrane composition, or an increase in fatty acid synthesis. It could also be due to a change in the degree of saturation of fatty acid side chains. As mentioned above, ^1^H MR spectra of intact Jurkat cells treated with Fas mAb showed an increase in the CH_2_/CH_3_ ratio relative to control indicating a possible increase in lipid mobility and/or fatty acid synthesis. ^1^H MR spectra of cell extracts were thus investigated to learn more about the nature of the observed changes. Extraction destroys the supramolecular organisation of all lipids in the cells including the association between cholesterol esters and TAGs and makes it impossible to distinguish between those which were mobile and MR-visible in the intact cells and those which were immobilised, for example in the bilayer, and thus MR-invisible. ^1^H MR spectra of the lipid fractions of control and treated cell extracts were therefore used to investigate whether treatment with Fas mAb had led to an increase in fatty acid synthesis. Spectra of lipid fractions of control and treated cell extracts showed that the CH_2_ and CH_3_ peak areas as well as CH_2_/CH_3_ ratio were constant, suggesting that the total amount and composition of fatty acids within the cells did not change with the onset of apoptosis. An increase in CH_2_ and CH_3_ has been observed previously, and was associated with an increase in polyunsaturated fatty acid concentration (Hakumaki *et al*, 1999). In contrast, in our cells no change in the degree of unsaturation of fatty acid chains was detected as indicated by the peaks resonating at 2.8 and 5.4 p.p.m.

In our model, onset of apoptosis and alterations in CH_2_/CH_3_ ratio were associated with an increase in mobile TAG concentration. Confocal microscopy images of cells stained with the lipophilic dye Nile Red demonstrated that neutral lipids detected by ^1^H MRS could originate from lipid droplets that are located in the cell cytoplasm. Results of fluorescence intensity of Nile Red stained neutral lipids obtained by flow cytometry and the increase in the concentration of TAGs detected by ^1^H MRS were within experimental error, suggesting that TAG accumulation in Jurkat T-cells originates mostly from cytoplasmic lipid droplets. This is in agreement with results from others reporting diffusion coefficient values for lipid signals in apoptotic cells which are consistent with lipids present in cytoplasmic droplets rather than in restricted membrane domains (Hakumaki *et al*, 1999).

Prior studies have shown that the prominent neutral lipid signals detected by ^1^H MRS can be due to cell cycle arrest caused by saturation density or acidic pH (Delikatny *et al*, 1996; Barba *et al*, 1999). Others have speculated that accumulation of lipids is possibly due to cells undergoing growth arrest prior to apoptosis subsequent to gene therapy (Hakumaki *et al*, 1999). This study shows no accumulation of cells in a particular phase of the cell cycle, suggesting that the neutral lipids detected in our ^1^H MR spectra of Jurkat cells are correlated only with apoptosis.

It is also interesting to note that the accumulation of TAGs was first detected at 1 h following the stimulation of the Fas pathway which is as early as the loss of the mitochondrial ΔΨ_m_ and prior to the appearance of the hypodiploid cells which occurred at 3 h following Fas mAb treatment. This indicates that Fas-induced apoptosis correlates with a relatively early increase in TAGs.

Regarding the source of the accumulated TAGs, ^1^H MRS-detected neutral lipid accumulation could result from an increase in fatty acid synthesis and subsequent accumulation of TAGs. However, this can be ruled out by the fact that no increase in the CH_2_ and CH_3_ peak areas was detected in spectra of cell extracts.

^1^H MRS-detected neutral lipid accumulation has also been suggested to result from impairment in the PtdCho turnover pathway either by activated PtdCho catabolism, or by inactivated PtdCho anabolism (Veale *et al*, 1996). This is in line with our observation of a significant drop in PtdCho. Considering the PtdCho anabolic pathway, as the cells are preparing to die, they could be reducing their uptake of choline, hence less PtdCho would be synthesised. This would lead to an increase in the other PtdCho substrate DAG, which could then be converted to TAG by the cells to prevent disruption of cellular metabolism (Exton, 1994). Others have reported an inhibition of PtdCho synthesis at the level of cytidine diphoshate-choline:1,2-diacylglycerol choline phosphotransferase (CPT) following intracellular acidification (Williams *et al*, 1998; Anthony *et al*, 1999). This could be the cause for the accumulation of TAGs in our cells.

Considering the PtdCho catabolic pathway, different phospholipases have been reported to be activated during Fas-induced apoptosis including PtdCho-specific phospholipase C (Cifone *et al*, 1995), phospholipase A_2_ (Atsumi *et al*, 1998) and phospholipase D (Han *et al*, 1999). The increase in lipids associated with HSV-tk-induced apoptosis was attributed to the hydrolysis of PtdCho into lysophosphatidylcholine and free fatty acids via the activation of phospholipase A_2_ (Hakumaki *et al*, 1999).

Lysophosphatidylcholine is below the detection level in our control and treated cell spectra. Hence some increase in lysophosphatidylcholine could remain unobserved in our system. Lysophosphatidylcholine could also be hydrolysed into fatty acids and glycerophosphocholine (GPC). However, we have found that GPC dropped rather than increased in our treated cells (data not shown), indicating that this catabolic pathway is unlikely in our system. Other groups have suggested that ^1^H MRS detectable mobile lipids might be generated by the catabolism of PtdCho via the action of PtdCho-specific phospholipase C activity (Veale *et al*, 1997; Ferretti *et al*, 1999). In our system this is not very likely because phosphocholine drops following treatment (Al-Saffar *et al*, 1999). Further work is required to determine whether the PtdCho depletion observed in our cells is linked to the accumulation of TAGs described here.

In summary, we have demonstrated that apoptosis correlates with an increase in CH_2_/CH_3_ ratio as well as an accumulation of TAGs in cytoplasmic lipid droplets. This event is specific to apoptosis and occurs as early as the loss of mitochondrial ΔΨ_m_ and hence could be a useful indicator of apoptotic cell death in treated tumours. These findings are also being extended to chemotherapy induced apoptosis.

## References

[bib1] Al-SaffarNMClarkePADiStefanoFLeachMORonenSM1999 Detection of metabolic changes associated with Fas- and chemotherapy-induced apoptosis using MRS Proc ISMRM(Abstract)

[bib2] AnthonyMLZhaoMBrindleKM1999 Inhibition of phosphatidylcholine biosynthesis following induction of apoptosis in HL-60 cells J Biol Chem 274 1968619692 10391908 10.1074/jbc.274.28.19686

[bib3] AtsumiG-ITajimaMHadanoANakataniYMurakamiMKudoI1998 Fas-induced arachidonic acid release is mediated by Ca2+-independent phospholipase A2 but not cytosolic phospholipase A2, which undergoes proteolytic inactivation J Biol Chem 273 1387013877 9593733 10.1074/jbc.273.22.13870

[bib4] BarbaICabañasMEArusC1999 The relationship between nuclear magnetic resonance-visible lipids, lipid droplets, and cell proliferation in cultured C6 cells Cancer Res 59 18611868 10213493

[bib5] BarbaIMannPCabañasMEArusCGasparovicC2001 Mobile lipid production after confluence and pH stress in perfused C6 cells NMR in Biomedicine 14 3340 11252038 10.1002/nbm.688

[bib6] BhakooKKBellJD1997 The application of NMR spectroscopy to the study of apoptosis Cell Mol Biol 43 621629 9298586

[bib7] BlankenbergFGKatsikisPDStorrsRWBeaulieuCSpielmanDChenJYNaumovskiLTaitJF1997 Quantitative analysis of apoptotic cell death using proton nuclear magnetic resonance spectroscopy Blood 89 37783786 9160684

[bib8] BlankenbergFGStorrsRWNaumovskiLGoralskiTSpielmanD1996 Detection of apoptotic cell death by proton nuclear magnetic resonance spectroscopy Blood 87 19511956 8634443

[bib9] CasuMAndersonGJChoiGGibbonsWA1991 NMR lipid profile of cells, tissues and body fluids: I-1D and 2D proton NMR of lipids from rat liver Magn Reson Chem 29 594602

[bib10] CifoneMGRoncaioliPDe MariaRCamardaGSantoniARubertiGTestiR1995 Multiple pathways originate at the Fas/APO-1 (CD95) receptor: Sequential involvement of phosphatidylcholine-specific phospholipase C and acidic sphingomyelinase in the propagation of the apoptotic signal EMBO J 14 58595868 8846779 10.1002/j.1460-2075.1995.tb00274.x PMC394704

[bib11] DelikatnyEJLanderCMJeitnerTMHancockRMountfordCE1996 Modulation of MR-visible mobile lipid levels by cell culture conditions and correlations with chemotactic response Int J Cancer 65 238245 8567123 10.1002/(SICI)1097-0215(19960117)65:2<238::AID-IJC18>3.0.CO;2-9

[bib12] Di VitoMLentiLKnijnAIorioEd'AgostinoFMolinariACalcabriniAStringaroAMeschiniSAranciaGBozziAStromRPodoF2001 H-1 NMR-visible mobile lipid domains correlate with cytoplasmic lipid bodies in apoptotic T-lymphoblastoid cells Biochim Biophy Acta-Mol Cell Biol Lipids 1530 4766 10.1016/s1388-1981(00)00165-7 11341958

[bib13] EllisPASmithIEMcCarthyKDetreSSalterJDowsettM1997 Preoperative chemotherapy induces apoptosis in early breast cancer [4] Lancet 349 849 9121265 10.1016/s0140-6736(05)61752-7

[bib14] EngelmannJHenkeJWillkerWKutscherBNossnerGEngelJLeibfritzD1996 Early stage monitoring of miltefosine induced apoptosis in KB cells by multinuclear NMR spectroscopy Anticancer Res 16 14291439 8694511

[bib15] ExtonJH1994 Phosphatidylcholine breakdown and signal transduction Biochim Biophy Acta – Lipids & Lipid Metabol 1212 2642 10.1016/0005-2760(94)90186-4 8155724

[bib16] FerrettiAKnijnAIorioEPulcianiSGiambenedettiMMolinariAMeschiniSStringaroACalcabriniAFreitasIStromRAranciaGPodoF1999 Biophysical and structural characterization of 1H-NMR-detectable mobile lipid domains in NIH-3T3 fibroblasts Biochim Biophy Acta-Mol Cell Biol Lipids 1438 329348 10.1016/s1388-1981(99)00071-2 10366776

[bib17] GreenDR2000 Apoptotic pathways: Paper wraps stone blunts scissors Cell 102 14 10929706 10.1016/s0092-8674(00)00003-9

[bib18] GreenspanPMayerEPFowlerSD1985 Nile red: A selective fluorescent stain for intracellular lipid droplets J Cell Biol 100 965973 3972906 10.1083/jcb.100.3.965 PMC2113505

[bib19] HakumakiJMPoptaniHSandmairA-MYla-HerttualaSKauppinenRA1999 1H MRS detects polyunsaturated fatty acid accumulation during gene therapy of glioma: Implications for the in vivo detection of apoptosis Nature Med 5 13231327 10546002 10.1038/15279

[bib20] HanJ-SHyunB-CKimJ-HShinI1999 Fas-mediated activation of phospholipase D is coupled to the stimulation of phosphatidylcholine-specific phospholipase C in A20 cells Arch Biochem Biophys 367 233239 10395739 10.1006/abbi.1999.1250

[bib21] HickmanJA1996 Apoptosis and chemotherapy resistance Euro J Can 32 921926 10.1016/0959-8049(96)00080-9 8763333

[bib22] HotzMAGongJTraganosFDarzynkiewiczZ1994 Flow cytometric detection of apoptosis: Comparison of the assays of in situ DNA degradation and chromatin changes Cytometry 15 237244 8187583 10.1002/cyto.990150309

[bib23] KauppinenRANiskanenTHakumakiJMWilliamsSR1993 Quantitative analysis of ^1^H NMR detected proteins in the rat cerebral cortex *in vivo* and *in vitro* NMR in Biomedicine 6 242247 8217525 10.1002/nbm.1940060403

[bib24] RonenSMDiStefanoFMcCoyCLRobertsonDSmithTADAl-SaffarNMTitleyJCunninghamDCGriffithsJRLeachMOClarkePA1999 Magnetic resonance detects metabolic changes associated with chemotherapy-induced apoptosis Br J Cancer 80 10351041 10362112 10.1038/sj.bjc.6690459 PMC2363054

[bib25] VealeMFDingleyAJKingGFKingNJC1996 1H-NMR visible neutral lipids in activated T lymphocytes: Relationship to phosphatidylcholine cycling Biochim Biophy Acta – Lipids & Lipid Metabol 1303 215221 10.1016/0005-2760(96)00104-x 8908156

[bib26] VealeMFRobertsNJKingGFKingNJC1997 The generation of 1H-NMR-Detectable mobile lipid in stimulated lymphocytes: Relationship to cellular activation, the cell cycle, and phosphatidylcholine-specific phospholipase C Biochem Biophys Res Commun 239 868874 9367861 10.1006/bbrc.1997.7566

[bib27] WilliamsSNOAnthonyMLBrindleKM1998 Induction of apoptosis in two mammalian cell lines results in increased levels of fructose-1,6-bisphosphate and CDP-choline as determined by 31P MRS. Magn Reson Med 40 411420 10.1002/mrm.1910400311 9727944

